# The State of Antimicrobial Resistance of Gram-Negative Bacilli in Canada

**DOI:** 10.3390/tropicalmed10040115

**Published:** 2025-04-21

**Authors:** Jeremy Li, Andrew Walkty, Philippe Lagacé-Wiens, James Karlowsky, George Zhanel

**Affiliations:** 1 Department of Medical Microbiology and Infectious Diseases, Max Rady College of Medicine, University of Manitoba, Winnipeg, MB R3E 0J9, Canada; awalkty@sharedhealthmb.ca (A.W.); plagacewiens@sharedhealthmb.ca (P.L.-W.); jkarlowsky@sharedhealthmb.ca (J.K.); ggzhanel@pcsinternet.ca (G.Z.); 2 Shared Health, Winnipeg, MB R3A 1R9, Canada

**Keywords:** antimicrobial resistance, extended-spectrum beta-lactamases, carbapenemases, *Pseudomonas aeruginosa*, multi-drug-resistance, antimicrobial stewardship

## Abstract

In the last two decades, there has been an increase in resistance among Gram-negative bacteria in Canada. From 2007 to 2016, the proportion of ESBL-producing isolates among *Escherichia coli* and *Klebsiella pneumoniae* isolates increased from 3.5% to 11.1%. There has also been an increase in carbapenem use over this time period, which may be contributing to the increasing prevalence of carbapenemase-producing Enterobacterales (CPE) in Canada. CPE, which were historically associated with travel, are now mostly acquired domestically. The prevalence of multi-drug resistant (MDR) *Pseudomonas aeruginosa* has decreased slightly, possibly due to decreasing use of fluoroquinolones and aminoglycosides. Many of the most effective antimicrobials for the treatment of infections with resistant Gram-negative organisms, including many of the novel β-lactam/β-lactamase inhibitors (βL/βLIs), are not marketed in Canada. A coordinated focus on antimicrobial stewardship and infection control is necessary to slow the spread of resistance and to preserve the efficacy of our current antimicrobials for future generations.

## 1. Introduction

The global rise of antimicrobial resistance (AMR) over the last few decades is a pressing threat to human health and affects low-income and high-income countries alike. In 2019, it is estimated that more than 1.27 million deaths globally were attributable to bacterial AMR [1].

The spread of AMR throughout Canada is of great concern for Canadian patients, physicians, and governments. Infections with resistant Gram-negative bacilli are more difficult to treat, are often initially treated with ineffective empiric antimicrobial therapy, and are associated with a much higher risk of mortality [2,3,4]. It is estimated that in 2018, 14,000 deaths and CAD 1.4 billion in costs were associated with AMR in Canada [5].

Despite increases in the prevalence of resistant organisms worldwide, the pace of development of antimicrobials has slowed. From the 1980s to the early 2000s, approval of new antimicrobials declined by 90%. Few new classes of antimicrobials are being discovered, primarily due to economic, scientific, and technical hurdles [6]. Of the few novel antimicrobial agents that have been developed to treat antimicrobial-resistant infections, most of them are not available or approved for use in Canada. In the context of dwindling development and approval of new antimicrobials, the increasing prevalence of antimicrobial resistance in Gram-negative bacilli is of even greater concern.

In this review, we discuss trends in the rates of extended-spectrum beta-lactamase (ESBL) production among Enterobacterales, trends in the prevalence of carbapenem-resistant Enterobacterales-CRE (including carbapenemase producing Enterobacterales-CPE), and trends in the prevalence of multi-drug-resistant (MDR) *Pseudomonas aeruginosa*. We review the impact of these trends on the effectiveness of empiric antimicrobial regimens for clinical syndromes such as urinary infections and hospital-acquired pneumonias and discuss concerns regarding access to effective therapy for the treatment of resistant Gram-negative infections.

## 2. ESBL-Producing Enterobacterales in Canada

Extended-spectrum beta-lactamases (ESBLs) are enzymes that hydrolyze and confer resistance to oxyimino-cephalosporins, including ceftriaxone, cefotaxime, and ceftazidime. Ceftriaxone is widely used as empiric treatment for numerous infectious syndromes. Although early ESBL enzymes were variants of TEM and SHV beta-lactamases, CTX-M-type enzymes are now the most common ESBL worldwide and in Canada [7,8], predominantly due to the spread of ST131, a pandemic clone of *Escherichia coli* [9,10].

### 2.1. Prevalence of ESBL-Producing Enterobacterales in Canada

**Figure 1:**
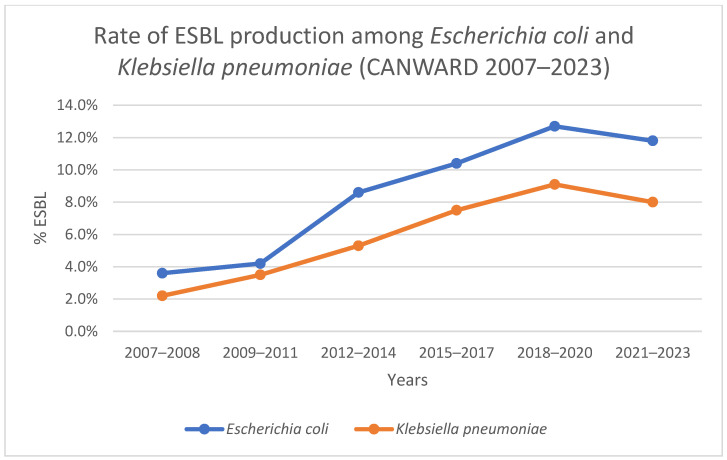
Rates of ESBL production among *Escherichia coli* and *Klebsiella pneumoniae* in Canada [11].

Trends in the prevalence of ESBL-producing bacteria in Canada can be inferred from the CANWARD study, an ongoing national surveillance study assessing the pathogens causing infections in Canadian hospitals and their susceptibility patterns. As part of the CANWARD study, hospitals across Canada submit a representative sample of their clinical isolates from respiratory, wound, urine, and blood cultures to the CANWARD coordinating laboratory for identification and antimicrobial susceptibility testing. Published data from the CANWARD study have shown that the proportion of *E. coli* isolates in Canada that produce ESBLs had increased rapidly, from 3.4% in 2007 to 11.1% in 2016. The proportion of *K. pneumoniae* isolates in Canada that produce ESBLs had also increased rapidly, from 1.3% in 2007 to 9.7% in 2016 [7]. More recent, unpublished data from the CANWARD study show that the rate of ESBL production among these organisms has been relatively stable since 2016, with a rate of ESBL production of 11.8% among *E. coli* and 8.0% among *K. pneumoniae* isolates, between 2021 and 2023 (Figure 1) [11]. The prevalence of ESBL-producing organisms varies markedly by specimen source, with the highest rates among respiratory isolates. The average prevalence of ESBL-producing isolates over the course of the 17-year study was 16.8% in respiratory specimens, 11.6% in wound and abscess specimens, 8.0% in blood specimens, and 6.3% in urine specimens. Canada’s Antimicrobial Resistance Network (AMRNet) reports that in 2020 the proportion of *E. coli* isolates from urine and blood that were non-susceptible to ceftriaxone was 18.5% and 21.2%, respectively, and the proportion of *K. pneumoniae* isolates from urine and blood that were non-susceptible to ceftriaxone was 5.1% and 11.8% respectively, noting that non-susceptibility to ceftriaxone may be mediated by mechanisms other than ESBL production [12].

### 2.2. Outcomes of ESBL-Producing Enterobacterales Infections

Ceftriaxone has been, for decades, the first-line antimicrobial for the empiric treatment of pyelonephritis [13,14,15], pneumonia [16], and intra-abdominal infections [17] and is widely recommended as the first-line treatment regimen for these syndromes by antimicrobial stewardship programs across Canada. In addition, piperacillin/tazobactam, a β-lactam/β-lactamase inhibitor with activity against *P. aeruginosa*, is widely used in Canada as the first-line empiric antimicrobial for the treatment of life-threatening sepsis [18,19], febrile neutropenia [20], and hospital-acquired and ventilator-associated pneumonias [16]. Although ESBLs are inhibited in-vitro by piperacillin/tazobactam, clinical studies suggest a higher rate of mortality when infections with ceftriaxone-resistant *E. coli* or *K. pneumoniae* are treated with piperacillin/tazobactam compared to carbapenems [21]. The rising prevalence of ESBLs increases the risk that patients will be initially treated with potentially ineffective antimicrobials, which is associated with treatment failure, complications, and higher mortality [22,23]. An observational study of 43 cases of *E. coli* bloodstream infections in Spain showed that while the empirical choice of antimicrobial treatment was determined to be adequate in every single case, it was still considered appropriate in only 51% of cases when in-vitro susceptibility test results were reviewed, due to the high prevalence of isolates producing CTX-M-15 beta-lactamase. This study also demonstrated that empirical therapy of ESBL-producing *E. coli* bacteremia with cephalosporins was associated with a mortality rate that is several times higher compared to treatment with a carbapenem or a β-lactam/β-lactamase inhibitor (35% vs. 9%) [23]. With the increasing prevalence of infections with ESBL-producing organisms among Canadian patients, these concerns are very pertinent to the Canadian context.

### 2.3. ESBL-Producing Enterobacterales Are MDR

A significant concern is that ESBL-producing Enterobacterales are frequently multi-drug resistant (MDR, defined as concomitantly resistant to ≥3 different antimicrobial classes). High rates (>50%) of non-susceptibility of both ESBL-producing *E. coli* and *K. pneumoniae* to trimethoprim-sulfamethoxazole (TMP-SMX) and fluoroquinolones such as ciprofloxacin and levofloxacin have been reported [24]. Among isolates collected for the CANWARD study from 2007 to 2023, 74.7% of ESBL-producing *E. coli* isolates and 85.1% of ESBL-producing *K. pneumoniae* isolates were MDR [11]. ESBL-producing *E. coli* and *K. pneumoniae* isolates are commonly resistant to TMP-SMX and ciprofloxacin. TMP-SMX and fluoroquinolones are frequently used as empiric therapy for urinary tract infections, directed therapy for wound infections, and as carbapenem-sparing agents in infections due to ESBL-producing organisms.

### 2.4. Treatment of ESBL-Producing Enterobacterales

The increasing prevalence of ESBL-producing organisms, many of which are also MDR, necessitates increasing use of carbapenems for the definitive treatment of infections. Moreover, concern among healthcare providers that ceftriaxone and piperacillin/tazobactam are no longer adequate for the empiric treatment of severe infections may be fuelling increasing and sometimes inappropriate empiric use of carbapenems [25]. Between 2015 and 2019, the use of carbapenems in Canada increased by 68% [26]. Rapidly increasing carbapenem use in Canada is likely to further drive selection pressure for carbapenem-resistance among Gram-negative organisms and may be contributing to the increasing prevalence of carbapenem-resistant Enterobacterales-CRE [27].

## 3. Carbapenem-Resistant Enterobacterales-CRE

Carbapenems are broad-spectrum antimicrobials that have historically been reserved for the directed treatment of infections caused by highly resistant Gram-negative organisms. The increasing prevalence of ESBLs has led to an increase in both directed and empirical use of carbapenems, which is leading to concerns about the emergence and increasing prevalence of carbapenem-resistant Enterobacterales (CRE). Receipt of broad-spectrum antimicrobials is the most frequently cited risk factor for subsequent infection with CRE [28].

### 3.1. Definitions of CRE

CRE encompass all Enterobacterales that are resistant to carbapenems, but the mechanism of resistance varies. CRE are categorised into carbapenemase-producing Enterobacterales (CPE) and non-CPE organisms [29,30,31]. Carbapenem resistance in non-CPEs are frequently due to expression of AmpC or ESBL beta-lactamases, in combination with alterations in porin channels or expression of efflux pumps, resulting in low-level carbapenem resistance (MICs 4–16 μg/mL) [32]. However, the more worrisome CRE are the CPE as they are often associated with high level (MICs ≥ 32 µg/mL) resistance to carbapenems [30,31]. Carbapenemase-producing Enterobacterales (CPEs) are of utmost concern for public health and infection control because carbapenemase genes are often carried on mobile genetic elements and may be transmitted between organisms. Dissemination of CPEs in hospitals and long-term care homes has resulted in serious and life-threatening nosocomial infections, as well as the rapid global expansion of carbapenem-resistance worldwide [31].

### 3.2. Outcomes of CRE and CPE Infections

Infections with CREs are associated with much higher mortality than those caused by non-CREs [2,3]. A multicentre prospective cohort study conducted between 2010 and 2014 in Italian onco-hematological patients found that the mortality rate of patients with carbapenem-resistant *K. pneumoniae* bloodstream infections was 52.2%, compared to 36.3% among those with susceptible *K. pneumoniae* infections [3]. A 2014–2018 retrospective study of 285 patients with *K. pneumoniae* bloodstream infection in China found that the 28-day mortality rate amongst patients with carbapenem-resistant *K. pneumoniae* infections was 50.0%, compared to 15.9% for those with susceptible infections [2]. Infections caused by CPEs are also associated with high mortality. In the United States, CPEs are associated with mortality rates as high as 40–50% [33,34], although most of these reports were from studies conducted prior to the availability of the newer β-lactam/β-lactamase inhibitors. Thirty-day all-cause mortality among Canadian patients with CPE infections has been reported at 16.3% (from 2018–2022) [35].

### 3.3. Prevalence of CRE and CPE in Canada

**Figure 2:**
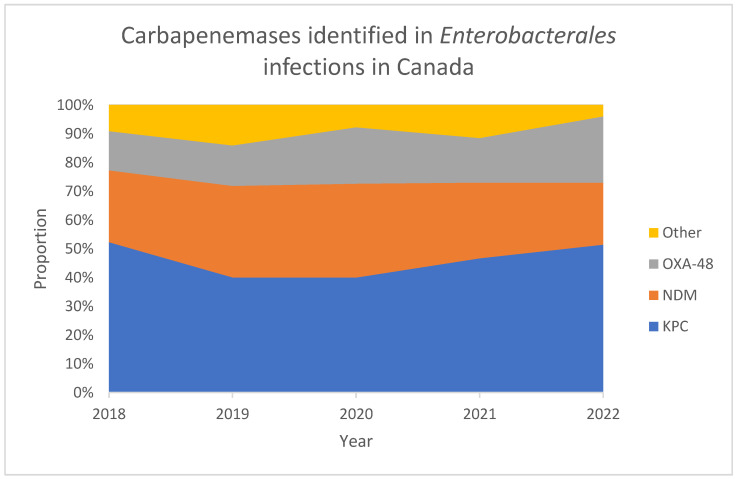
Proportion of Carbapenemases identified in Enterobacterales infections in Canada. NDM = New Delhi metallo-β-lactamase, KPC = *Klebsiella pneumoniae* carbapenemase, OXA-48 = OXA-48-like β-lactamase [35].

Historically, despite the alarming spread of CRE and particularly carbapenemase-producing *K. pneumoniae* in the United States in the 2000s, Canada has been relatively spared. However, a review of longitudinal surveillance data from the Canadian CANWARD study, from 2007 to 2016, found a small but significant increase in the frequency of carbapenem-resistant *K. pneumoniae* [24]. Unfortunately, this trend has continued. Data from the Canadian Nosocomial Infection Surveillance Program (CNISP) show that the incidence of nosocomial CPE infections has increased by an alarming 133% from 2018 to 2022, from 0.06 to 0.14 infections per 10,000 patient days [35]. The predominant carbapenemases identified in Canada were *Klebsiella pneumoniae* carbapenemase (KPC), New Delhi metallo-β-lactamase (NDM), and OXA-48-like β-lactamases (OXA-48) (Figure 2). These carbapenemases account for 90% of CPEs in Canada. Historically, CPE infections were mostly associated with international travel and receipt of healthcare outside of Canada [35]. However, in recent years, most cases of CPE infection have been associated with nosocomial acquisition in Canada. Of the 298 patients with CPE infections identified by CNISP from 2018 to 2022, only a minority (22.8%) were associated with travel outside of Canada [35]. These findings suggest that KPC, NDM, and OXA-48 may be becoming endemic in Canadian hospitals, and we should expect that rates of CPE colonization and infection will continue to increase.

### 3.4. CRE and CPE Are MDR

CPE isolates in Canada with NDM and KPC carbapenemases are frequently MDR, with resistance to β-lactam and non-β-lactam classes of antimicrobials including fluoroquinolones and trimethoprim-sulfamethoxazole. In 2022, 49.2% of NDM CPE isolates submitted to the Canadian Nosocomial Infection Surveillance Program (CNISP) were extensively drug resistant (XDR, defined as resistance to at least one agent in all but two or fewer antimicrobial classes), and an additional 45.8% were MDR. In 2022, 21.7% of KPC CPE isolates submitted to CNISP were XDR and an additional 44.1% were MDR [35].

### 3.5. Treatment of CRE and CPE

Ideally, infections with CPEs should be treated with the newer β-lactam/β-lactamase inhibitors (βL-βLIs) ceftazidime/avibactam, meropenem/vaborbactam, imipenem/relebactam or cefiderocol. Infections with metallo-β-lactamase-producing organisms should be treated with ceftazidime/avibactam in combination with aztreonam, or with cefiderocol [36]. Of these antimicrobials, meropenem/vaborbactam is the only medication approved for clinical use in Canada, having received Health Canada approval in December 2024. The remaining antimicrobials are only available through the Health Canada Special Access Program. These medications are therefore not on hospital formularies and may not be readily available in hospital pharmacies, which could lead to a delay in patients receiving effective antimicrobial treatment or result in the use of less effective, more toxic regimens, usually combinations of antimicrobials including meropenem with colistin and/or tigecycline and/or an aminoglycoside.

## 4. Multi-Drug Resistant (MDR) *Pseudomonas aeruginosa*

### 4.1. Definition of MDR P. aeruginosa

The US Centers for Disease Control (CDC) and European Centre for Disease Prevention and Control define multi-drug resistance (MDR) as non-susceptibility to at least one agent in 3 different classes of antimicrobials [37]. For *P. aeruginosa*, MDR is defined as non-susceptibility to at least one agent in 3 of the following classes: penicillins, cephalosporins, fluoroquinolones, aminoglycosides, and carbapenems [36].

### 4.2. Prevalence of MDR P. aeruginosa in Canada

**Figure 3:**
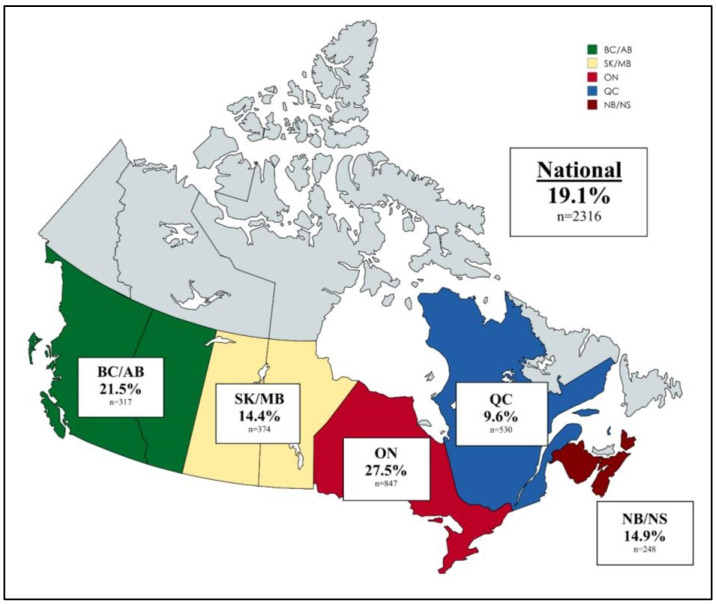
Regional prevalence of MDR *Pseudomonas aeruginosa* in Canada (CANWARD 2014–2020). Adapted from Walkty et al. [39]. BC/AB = British Columbia and Alberta, SK/MB = Saskatchewan and Manitoba, ON = Ontario, QC = Quebec, NB/NS = New Brunswick and Nova Scotia.

The CANWARD national surveillance study, conducted between 2008 and 2015, showed that the rate of MDR *P. aeruginosa* isolates in Canada was 14.5% over the 8-year period [38]. Regional data of MDR *P. aeruginosa* have been reported at 9.6–27.5% (Figure 3) [39]. There was a small but statistically significant decrease in the rate of MDR isolates over time. MDR isolates were most frequently recovered from patients in an ICU setting or on a medical ward and were more frequently recovered from a respiratory site of infection compared to urine and wound sources. Gentamicin and colistin remained the most active agents. The decreasing rate of MDR *P. aeruginosa* is predominantly driven by increasing susceptibility to ciprofloxacin, gentamicin, and colistin [38]. This may be due to decreasing use of these antimicrobials as a result of antimicrobial stewardship efforts or changes in prescribing practices in favour of antimicrobials with less toxicity. For example, from 2008 to 2022, there has been a 50% decrease in the rate of outpatient prescriptions for fluroquinolones [40]. This decrease may be related to a 2017 safety review of fluoroquinolones by Health Canada, which resulted in a risk communication to Canadian health professionals and a safety warning added to fluroquinolone monographs in Canada [41].

In 2018, Kadri et al. introduced the concept of *P. aeruginosa* with difficult-to-treat resistance (DTR) [42]. Recognizing that some antibiotic classes are more toxic and less preferred than others, DTR is defined as *P. aeruginosa* resistant to all tested beta-lactams, fluroquinolones, and carbapenems. Moreover, gentamicin breakpoints were deleted by the Clinical and Laboratory Standards Institute (CLSI) in 2023 [43]. There has not been an updated analysis of *P. aeruginosa* resistance in Canada since 2015. An updated study is necessary and should ideally use the DTR definition of resistance.

### 4.3. Resistance Mechanisms of MDR P. aeruginosa in Canada

Among a representative sample of 206 Canadian isolates of carbapenem-resistant *P. aeruginosa* isolates collected in 2009–2010, only 11 were carbapenemase-producing, as follows: *bla*_GES-5_ (*n* = 3), *bla*_VIM-4_ (*n* = 7), and *bla*_VIM-2_ (*n* = 1) [44]. Most carbapenemase-resistant isolates of *P. aeruginosa* were not carbapenemase-producing. Resistance to carbapenems in non-carbapenemase-producing *P. aeruginosa* can be attributed to efflux, deficiency in the OprD porin, or increased AmpC production [45].

### 4.4. Treatment of MDR P. aeruginosa

Carbapenem-resistant *P. aeruginosa* are frequently MDR [44], limiting treatment options. For *P. aeruginosa* not susceptible to any of the usual first-line anti-pseudomonas antimicrobials, ceftolozane/tazobactam, ceftazidime/avibactam, imipenem/relebactam, and cefiderocol are treatment options [36]. For metallo-beta-lactamase producing strains of *P. aeruginosa* resistant to other first-line antimicrobials, cefiderocol is recommended and other βL/βLIs are unlikely to be effective [36]. Of these medications, only ceftolozane/tazobactam is approved by Health Canada. This may result in delay in therapy for patients with MDR *P. aeruginosa* infections.

## 5. Conclusions

Overall, there has been a concerning increase in the rate of ESBL-production among *Escherichia coli* and *Klebsiella pneumoniae* isolates in Canada. These isolates are resistant to 3rd-generation cephalosporins such ceftriaxone, which are commonly prescribed for urinary infections, pneumonia, and intra-abdominal infections. As ESBL-producing infections are frequently MDR to a variety of different antimicrobial classes, they are best treated with a carbapenem.

Though carbapenem-resistant Enterobacterales (CRE) infections remain uncommon in Canada, they are trending up. Carbapenemase-producing Enterobacterales (CPE), which were historically associated with international travel, are now mostly acquired domestically. This may signify that CPEs are becoming endemic in Canadian hospitals. The rapidly increasing rate of carbapenem use in Canada, often for the empiric treatment of serious infections, is concerning and is likely to further drive increasing carbapenem resistance. Canada currently does not have clinical access to the new β-lactam/β-lactamase inhibitors (βL/βLIs) (except meropenem/vaborbactam, which was recently approved by Health Canada) needed to treat CRE infections. In line with this, Health Canada has developed a national list of reserve antimicrobial drugs, modelled after the WHO AWaRe classification system of antimicrobial drugs, recommending that these antimicrobials be reserved as last resort treatments [46]. This list includes all carbapenems approved for use in Canada.

Infections caused by MDR *Pseudomonas aeruginosa* are common but relatively stable in Canada. However, there has not been an updated analysis of *P. aeruginosa* resistance in Canada since 2015. Recognizing that some antibiotic classes are more preferred than others, an updated analysis of *P. aeruginosa* resistance should use the difficult-to-treat resistance (DTR) definition of drug resistance. *P. aeruginosa* not susceptible to usual first-line agents will require treatment with ceftolozane/tazobactam, ceftazidime/avibactam, imipenem/relebactam, or cefiderocol. Of these, only ceftolozane/tazobactam is marketed in Canada.

A coordinated focus on antimicrobial stewardship and infection prevention and control is necessary to reduce the spread of resistant Gram-negative bacilli and preserve the efficacy of our current last-line antimicrobials for future patients in Canada. Additionally, it is imperative for pharmaceutical companies to work with Health Canada to bring newer βL/βLI agents, as well as cefiderocol, to the Canadian market.

## Data Availability

No new data were created or analyzed in this study.
